# Power Doppler Ultrasound Phenotyping of Expanding versus Collapsed Popliteal Lymph Nodes in Murine Inflammatory Arthritis

**DOI:** 10.1371/journal.pone.0073766

**Published:** 2013-09-09

**Authors:** Echoe M. Bouta, Yawen Ju, Homaira Rahimi, Karen L. de Mesy-Bentley, Ronald W. Wood, Lianping Xing, Edward M. Schwarz

**Affiliations:** 1 Center for Musculoskeletal Research, University of Rochester School of Medicine and Dentistry, Rochester, New York, United States of America; 2 Department of Biomedical Engineering, University of Rochester School of Medicine and Dentistry, Rochester, New York, United States of America; 3 Department of Pathology and Laboratory Medicine, University of Rochester School of Medicine and Dentistry, Rochester, New York, United States of America; 4 Department of Pediatrics, University of Rochester School of Medicine and Dentistry, Rochester, New York, United States of America; 5 Department of Obstetrics and Gynecology, University of Rochester School of Medicine and Dentistry, Rochester, New York, United States of America; 6 Department of Urology, University of Rochester School of Medicine and Dentistry, Rochester, New York, United States of America; Veterans Affairs Medical Center, United States of America

## Abstract

Rheumatoid arthritis is a chronic inflammatory disease manifested by episodic flares in affected joints that are challenging to predict and treat. Longitudinal contrast enhanced-MRI (CE-MRI) of inflammatory arthritis in tumor necrosis factor-transgenic (TNF-Tg) mice has demonstrated that popliteal lymph nodes (PLN) increase in volume and contrast enhancement during the pre-arthritic “expanding” phase of the disease, and then suddenly “collapse” during knee flare. Given the potential of this biomarker of arthritic flare, we aimed to develop a more cost-effective means of phenotyping PLN using ultrasound (US) imaging. Initially we attempted to recapitulate CE-MRI of PLN with subcutaneous footpad injection of US microbubbles (DEFINITY®). While this approach allowed for phenotyping via quantification of lymphatic sinuses in PLN, which showed a dramatic decrease in collapsed PLN versus expanding or wild-type (WT) PLN, electron microscopy demonstrated that DEFINITY® injection also resulted in destruction of the lymphatic vessels afferent to the PLN. In contrast, Power Doppler (PD) US is innocuous to and efficiently quantifies blood flow within PLN of WT and TNF-Tg mice. PD-US demonstrated that expanding PLN have a significantly higher normalized PD volume (NPDV) versus collapsed PLN (0.553±0.007 vs. 0.008±0.003; p<0.05). Moreover, we define the upper (>0.030) and lower (<0.016) quartile NPDVs in this cohort of mice, which serve as conservative thresholds to phenotype PLN as expanding and collapsed, respectively. Interestingly, of the 12 PLN phenotyped by the two methods, there was disagreement in 4 cases in which they were determined to be expanding by CE-MRI and collapsed by PD-US. Since the adjacent knee had evidence of synovitis in all 4 cases, we concluded that the PD-US phenotyping was correct, and that this approach is currently the safest and most cost-effective in vivo approach to phenotype murine PLN as a biomarker of arthritic flare.

## Introduction

Rheumatoid arthritis (RA) is a debilitating immune-mediated inflammatory disorder characterized by recurrent arthritic flares that lead to joint inflammation and destruction, and cause significant morbidity in RA patients [1]. Murine models of chronic RA, such as the TNF transgenic (TNF-Tg) mouse [2], [3], have proven useful in elucidating the pathophysiology of inflammatory-erosive arthritis and evaluating novel interventions. Contrast enhanced (CE)-MRI has emerged as a longitudinal outcome measure to quantify synovial and draining lymph node volume in murine models of inflammatory arthritis [4]–[11]. These studies have found that TNF-Tg mice with frank ankle arthritis had both larger popliteal lymph node (PLN) volume (PLNvol) and greater LN contrast enhancement (LNCE) when compared to their wild type (WT) littermates. To quantify this as a metric, LN capacity (LNCap  =  LNCE*PLNvol) was developed as a primary outcome measure to study PLN as a biomarker of inflammatory arthritis in the lower limbs of mice [4]. Subsequent CE-MRI studies demonstrated that arthritic knee flare was associated with the expansion and subsequent collapse of the PLN [8], [9], [11]. Thus, formal associations between altered PLNvol, LNCE and the onset of arthritic flare have been established.

MRI is commonly used to study synovium, tendons, bone and LN in RA pathogenesis [12]–[15]. By quantifying the amount of synovium and synovial fluid in the joint, CE-MRI can be used to identify patients with early RA [16], [17]. PLN have also been used as a biomarker because PLN in RA patients are usually larger in size than that of OA patients [18]. Over the last three decades, MRI has become the clinical imaging standard to aid in the diagnosis of RA and assess soft tissue and joint damage in RA patients. However, the high cost (machine time and labor) and limited access to these large instruments prevent MRI from being a useful tool to study inflammatory arthritis in animal models.

Compared to other musculoskeletal imaging modalities (i.e. MRI), ultrasound (US) has several remarkable advantages, incuding real-time imaging, easy accessiblity, cost-efficiency, and absense of ionizing radiation [19]. The use of US in RA patients provides high-resolution images of joints and surrounding tissues, and can be performed and interpreted by a rheumatologist in real-time, becoming an increasingly common imaging method in most rheumatology departments [20]. To assess synovitis and degree of joint damage in RA, both gray scale US and power Doppler (PD) US have proven to be useful [21]–[26]. The scoring system for gray scale US, which ranks the synovitis as normal, minor, moderate and severe synovitis, is generally accepted for semi-quantifiable purposes [23]. Similarly, gray scale or PD-US can also be used to evaluate tenosynovitis [27] and bone erosion [24], [28]. Two sets of semi-quantifiable scoring systems have been established for PD-US, which visualizes blood flow, using either the area of the PD signal [24] or the maximal degree of PD activity [22]. CE-US has also been employed in RA diagnosis, which has greater sensitivity versus PD-US at detecting vascularity and synovitis or tenosynovitis [29], [30].

Consistent with MRI findings, PLN changes in RA patients and mouse models of RA have been observed via US. It was previously found that there is a significant relationship between the PLNvol as measured by both MRI and US, showing an agreement between the two modalities [31]. Furthermore, the PD signal in draining LN of RA patients correlates with disease activity, and US is able to detect differences in cortical hypertrophy in these draining LN [32].

With the clinical success in RA patients, US has been used in animal models of RA [33], [34]. A scoring system based on gray scale and PD-US on knees and ankles in the collagen-induced arthritis (CIA) mouse model has been established with a strong correlation between histology and US score [33]. US has been used to evaluate synovitis in the antigen-induced arthritis (AIA) rabbit model to measure capsule thickness and CE-US to measure synovium thickness, which was found to significantly and positively correlate with the histological scoring of synovitis [35]. Both studies demonstrate the feasibility of US as an outcome measure of arthritis in small animals. Thus, in order to expand the utility of US to assess inflammatory arthritis in mice, we performed studies to establish an US measurement that can faithfully phenotype expanding versus collapsed PLN in TNF-Tg mice as a biomarker of arthritic flare in knee joints.

## Materials and Methods

### Ethics Statement

The research was conducted with approval by the University of Rochester Institutional Animal Care and Use Committee.

### Animals

TNF-Tg mice (the 3647 line) [2] were originally obtained from Dr. G. Kollias, and are maintained as heterozygotes in a C57BL/6 background. The first signs of inflammatory arthritis in these mice appear in the ankle joints at 2–3 months of age and progress to the knee at 4–7 months of age.

### CE-MRI and Analysis

MRI scans were performed in a 3T Siemens Trio (Siemens MedicalSolutions, Erlangen, Germany) as described previously [4], [5], [36]. Briefly, TNF-Tg mice were anesthetized with intraperitoneal ketamine (60 mg/kg) and xylazine (4 mg/kg) and the knee and ankle were inserted into a customized knee and ankle coil. After a pre-contrast MRI scan, gadolinium-diethylenetriamine pentaacetic acid (Gd-DTPA) contrast agent (Omniscan, Amersham Health, Oslo, Norway) was injected via orbital venous plexus at 0.5 mL/kg. The post-contrast scan was started 5 minutes after injection to allow for circulation of Gd-DTPA.

CE-MRI was used to measure knee synovial volume, PLNVol, and LNCE. Amira (VSG, Burlington, MA) was used to qualify these parameters as described previously [4], [5], [36]. Briefly, the 3D stack of pre-contrast scan was aligned with post-contrast via automatic registration. Then, a stack of images was generated by subtracting the pre-contrast scan from the post-contrast scan using the Arithmetic module. PLN and synovial volumes were segmented by manually drawing region of interests (ROIs) on the 3D stack. The knee synovial volumes were quantified as voxels above the threshold of 3.5 times the muscle mean signal intensity. The delineation of the PLN from the surrounding fat pad tissue was determined based on signal intensity >1500 arbitrary units. LNCE was defined as the LN signal intensity divided by muscle signal intensity.

### Ultrasound of PLN

Each PLN was imaged with a high-resolution small-animal ultrasound system (VisualSonics 770, Toronto, Ontario, Canada) using a 704b scanhead. Each mouse was anesthetized with 1.5% isoflurane in oxygen and hair removed from ankles to hips using a depilatory cream. The mouse was then placed in the supine position on a 37^°^C heated imaging platform with paws taped to surface electrodes for heart rate monitoring and respiratory cycle triggered image synchronization.

### US contrast studies

**Figure 1:**
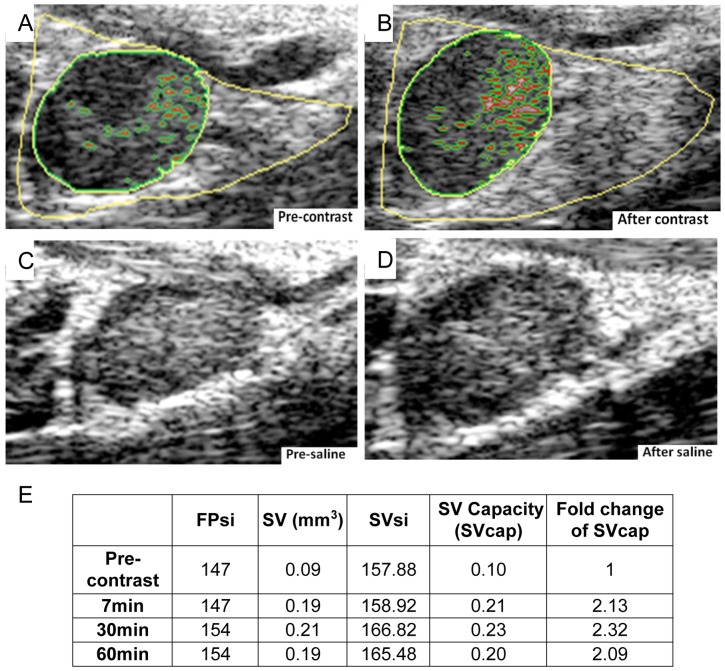
Quantification of PLN sinus volume capacity (SVcap) using CE-US. Representative 2D US images of an expanding PLN in a 4-Tg mouse were obtained before (A) and 30 min after DEFINITY® contrast inject (B) are shown to illustrate the method for quantifying PLN sinus volume (SV). First the PLN (dark tissue highlighted with green circle) within the fat pad (bright triangular tissue with yellow highlight) were identified in the region of interest (ROI). Then the SV within the PLN (red highlighted regions) was quantified by identifying the voxels within the PLN whose signal was greater than the mean fat pad signal intensity (FPsi). This was also performed on a 4 month old TNF-Tg mouse before (C) and after (D) saline injection into the afferent footpad, and the 2D images are shown without highlights. Note that pre-contrast SV (A) is expanded following DEFINITY® injection (B), which is readily visible due to its echogenic signal. In contrast, the increased SV could not be quantified following saline injection, which decreased US signal throughout the LN. (E) FPsi (arbitrary units; a.u.), SV (mm^3^), SV signal intensity (SVsi; a.u.), and SV Capacity (SVcap; a.u.) were calculated for the DEFINITY^®^ injected mouse with Amira as described in Materials and Methods. To normalize the SV to changes in FPsi following injection, the SVcap was calculated as (SV*SVsi)/FPsi. Note the 2-fold increase in SVcap at all time points following contrast injection.

When phenotyping collapsed versus expanding PLN using CE-US, TNF-Tg mice and WT controls (3 to 9 months of age) were anesthetized, then 3D images of the PLN and surrounding triangular fat pad were acquired before and 7, 30 and 60 minutes after a 50 μL subcutaneously injection of saline or DEFINITY® (Lantheus Medical Imaging, N. Billerica, MA) into the footpad. Analysis was performed using Amira. To segment and quantify PLNvol, ROIs of PLN and the surrounding fat pad were selected manually as described in Figure 1. The mean signal intensity of the fat pad (FPsi) was computed using the Tissue Statistics module. To eliminate the fat pad from the PLN, the areas in which the signal intensity was over FPsi were subtracted and any resultant empty inclusions within the PLN were filled. Changes of FPsi in a given mouse before and after DEFINITY® injections were less than 10%, demonstrating the remarkably consistent echogenic signal of this tissue, and its appropriateness for normalization in longitudinal studies. Thus, FPsi was used as a threshold value to quantify sinus volume (SV) (Figure 1A–B). Sinuses in the PLN were segmented to derive the LN SV at all time points, which were calculated based on the volume of contrast enhancement before and after DEFINITY^®^ injection. The time course study showed that SV reached the maximal value at 30 minutes after the injection (Figure 1E). The SV was normalized to FPsi, and sinus volume capacity (SVcap) was defined as SV *SVsi/FPsi.

### Power Doppler studies

**Figure 2:**
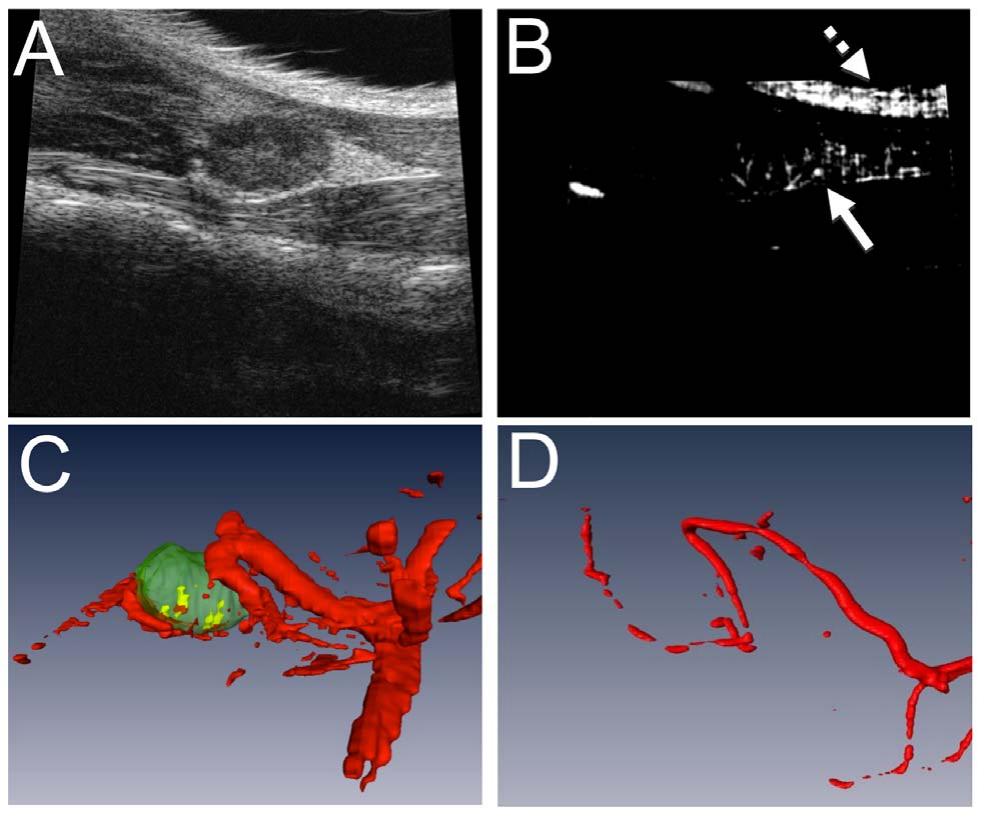
Quantification of normalized power Doppler volume (NPDV) using PD-US and validation with vascular micro-CT. Data from a representative 4-month-old TNF-Tg mouse is shown. PD-US was performed as described in Materials and Methods to generate the 2D images obtained using B mode (A) and PD (B) scans of the ROI of the knee including the fatpad in which the PLN resides. Note the artifact in the PD recording due to the surface of the skin (dashed arrow in B) versus the true PD signal (solid arrow in B). Images were then imported in Amira where the LN was manually segmented and the PD signal was thresholded to create 3D volumes. The PLNvol (green structure in C) was used to apply a mask to the total blood volume (red structures in C) to give the total PD volume within the LN (yellow structure in C). To confirm this methodology, the mouse underwent Microfil perfusion for vascular micro-CT analysis, and the 3D volume reconstruction is shown (D). Note the similarities in vessel structure with decreased vessel diameter due to live *in vivo* imaging vs perfusion.

TNF-Tg mice and their WT littermates (ages 3–9 months) underwent US imaging to detect the optimal area to perform PD. B mode (Figure 2A) and PD (Figure 2B) scans were performed of the entire fat pad in which the PLN resides with a wall filter of 2.5 mm/s, a scan speed of 2 mm/s, dynamic range of 13.13–24.06 dB and the number of pulses to radio-frequency (RF) cycles as two. After imaging was complete, data analysis was accomplished using Amira. First, the PLN was manually segmented as described above for US contrast studies, and the PD signal was thresholded (>64 arbitrary units; a.u.) to encompass the PD signal. Then, a mask was created by using the Arithmetic module, and the expression A*(B>0) was used, where A refers to the PD signal and B refers to the mask (PLN). This leaves a volume positive for Doppler signal that occupies the PLN (Figure 2C), which can be quantified via the Material Statistics module. The SurfaceGen and SurfaceView module were used to create 3D surfaces of these volumes. Then, the PD signal within the PLN was normalized by the volume of the PLN to derive the normalized PD volume (NPDV).

### Vascular micro-CT analysis

Lead chromate perfusion was performed as previously described [37]. Briefly, mice were anesthetized by intraperitoneal injection of ketamine (60 mg/kg) and xylazine (4 mg/kg) and a thoracotomy was performed to insert a 22G angiocatheter into the left ventricle. Then, 10 mL of PBS and heparin (100 IU/mL, Sigma, St. Louis, MO), followed by 10 mL of 4% paraformaldehyde (Sigma) were perfused into the left ventricle via the angiocatheter. This procedure was immediately followed by injection of 3 mL of the contrast agent MICROFIL MV-122 (Flow Tech, Inc., Carver, MA), a radiopaque silicone rubber compound containing lead chromate, via the same route. The PLN and surrounding tissue were stored in 4% paraformaldehyde at 4°C until the samples underwent micro-CT scan using a 10.5 micron isotropic high-resolution micro-CT (VivaCT 40; Scanco Medical AG, Basserdorf, Switzerland). To confirm that power Doppler is valid for vessels near the PLN, perfusion with Microfil was performed in the same animal that underwent PD-US. The same vessels that were seen with PD-US were found using micro-CT; the perfused vessels appear to have a smaller diameter due to differences in vital imaging versus perfusion (Figure 2D). Of note there was a limitation of Microfil to fill some of the smaller vessels that PD-US was able to detect (Figure2C–D).

### Electron Microscopy

The muscle with the afferent lymphatic vessel to the PLN was identified by injecting Evan's blue in the footpad and was then excised and fixed overnight at 4^°^C using a combination fixative of 2.5% glutaraldehyde and 4.0% paraformaldehyde in 0.1 M sodium cacodylate buffer. The specimens were rinsed in 0.1 M sodium cacodylate buffer and post-fixed with buffered 1.0% osmium tetroxide. The tissue was dehydrated in a graded series of ethanol to 100%, transitioned into propylene oxide, infiltrated with EPON/Araldite epoxy resin, followed by embedment in fresh resin and polymerization for 2 days at 70^°^C. To identify the lymphatic vessel in the specimen, the epoxy embedded block was cut serially into one micron slices and stained with Toluidine blue. Then the specimen was trimmed of excess surrounding tissue and thin sectioned at 70 nm with a diamond knife using an ultramicrotome. These thin sections were placed onto 150 mesh carbon coated nickel grids, and stained with uranyl acetate and lead citrate. A Hitachi 7650 Transmission Electron Microscope with a Gatan 11 megapixel Erlangshen digital camera was used to image the grids.

### Statistical analysis

Data was first assessed for normality and groups were checked to have equal variances by the Kolmogorov-Smirnoff test and F-test, respectively, if possible. Comparisons between groups were analyzed by a Kruskal-Wallis nonparametric one-way ANOVA with Dunn's post hoc test or two-sided t-test. *p* values less than 0.05 were considered significant.

## Results

**Figure 3:**
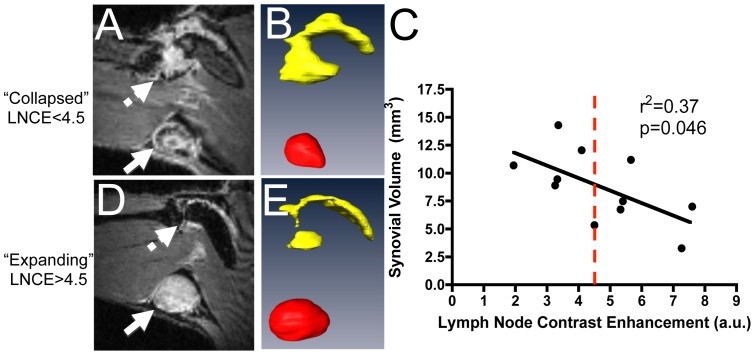
Lymph node contrast enhancement (LNCE) correlates with synovial volume and can be used to phenotype TNF-Tg mice. TNF-Tg mice (ages 6–12 months) underwent CE-MRI. Mice with a low LNCE (solid arrow) show a large synovial volume (dashed arrow) (A), while PLN with higher LNCE (solid arrow in B) correlate with less synovial volume (dashed arrow in D). PLN (red) and synovial volume (yellow) renditions were completed in Amira from the CE-MRI images, and are shown in B and E. Note the significant correlation between synovial volume and LNCE (n = 11) (C). From these data, a threshold of 4.5 was established to phenotype the PLN of TNF-Tg mice (red dashed line); a LNCE above 4.5 defines PLN as “expanding”, while PLN falling below the 4.5 threshold are phenotyped as “collapsed”.

Previously, CE-MRI has been used to phenotype TNF-Tg mice as expanding or collapsed based on a strict LNCap threshold of 30 arbitrary units (a.u.) [9]. Although we have consistently found that PLN expansion and collapse in an individual leg can be observed by longitudinal CE-MRI in TNF-Tg mice [4], [8], [9], [11], our attempts to demonstrate a statistically significant difference in PLNvol in cross-sectional cohorts of expanding versus collapsed have been unsuccessful due to the great variability of this dynamic process and differences of PLN in different mice [9]. Therefore, LNCE alone can be used to phenotype PLN via CE-MRI (Figure 3). This approach is further supported by the significant correlation between synovial volume and LNCE (Figure 3C). However, LNCE phenotyping of PLN as a method to randomize a cohort of arthritic mice into a prospective study suffers from the finding that values are a continuum as the PLN progress from expanding to collapsed. Thus, the LNCE threshold of 4.5 a.u. used to phenotype PLN seems somewhat arbitrary and inaccurate, as some PLN are very close to this value (Figure 3C). This concern, in addition to the very high costs of CE-MRI, warrants the development of a more cost-efficient method of phenotyping with more conservative threshold values for expanding and collapsed PLN.

**Figure 4:**
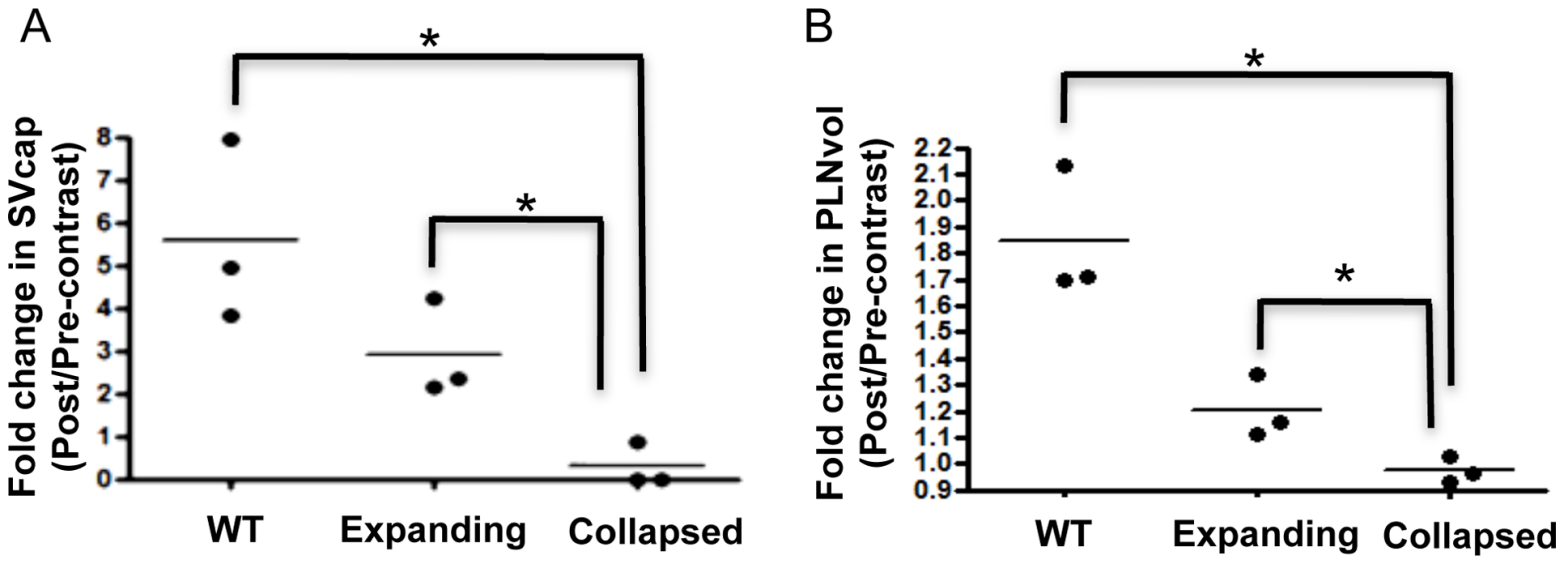
Differential effects of DEFINITY® injection on WT, expanding and collapsed PLN as evaluated by contrast enhanced ultrasound imaging. PLN in TNF-Tg mice were phenotyped as expanding or collapsed by CE-MRI as described in Figure 3, and then assessed by CE-US together with WT PLN from littermates as described in Figure 1. SVcap (A) and PLNvol (B) were determined for each PLN before and after DEFINITY^®^ injection, and the data are presented as the fold change for each PLN and the mean for each group (*p<0.05 vs. Collapsed Group by student t-test).

To determine if DEFINITY® contrast enhancement can be used to demonstrate the functional differences between expanding and collapsed PLN, US was performed before and after DEFINITY® injection into the footpads of WT and TNF-Tg mice. The results demonstrated a significant increase in SVcap (5.58-fold) and an 85% increase in PLNvol in WT mice (Figure 4). Similarly, DEFINITY® injection induced a 2.9-fold increase in SVcap and a 16% increase in PLNvol in expanding TNF-Tg PLN. Of note, expanding PLN in TNF-Tg mice are 2–5 times larger than PLN in WT mice, mostly due to increased fluid volume [4]. Thus, the limited ability for expanding PLN to increase in size following contrast injection suggests a saturated fluid volume (Figure 4B). In contrast, collapsed PLN did not show an increase in SVcap and PLN volume following afferent DEFINITY® injection, which is consistent with the very limited draining function [11], [38]. Importantly, our saline injections proved that the contrast enhancement is not due to the vehicle, and that injection of an echogenic media is required for this US imaging approach, as SVcap could not be calculated without contrast enhancement (Figure 1C–D).

**Figure 5:**
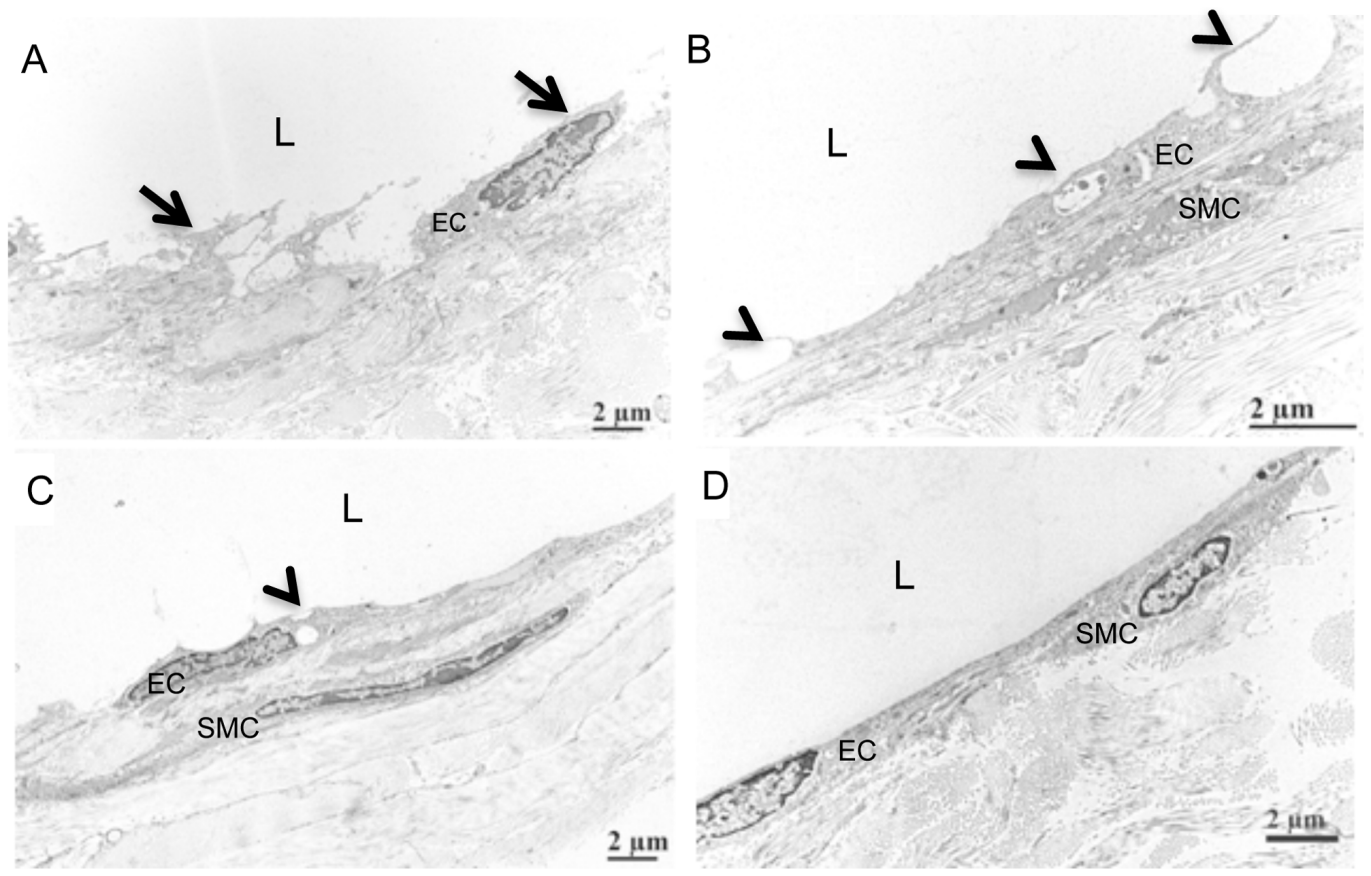
Lymphatic endothelial cell damage following afferent DEFINITY® injection. 4-month-old TNF-Tg and WT mice received a footpad injection of DEFINITY® or saline, and were subjected to US imaging as described in Materials & Methods. 4 weeks later, the injected footpad received an injection of Evan's blue dye to identify the lymphatic vessels afferent to the PLN, which were harvested and processed for transmission electron microscopy. Representative images are shown to illustrated the endothelial cells (EC); smooth muscle cells (SMC); and lumen (L) of the lymphatic vessel. Note the damaged lymphatic vessel in the DEFINITY^®^ injected mice (A,B), as evidenced by the cell detachment (arrows in A), and obviously large vacuoles and intraluminal protrusions (arrow heads in B), as well as the atrophic appearance of the smooth muscle cell (B). In contrast, the lymph vessels exposed to saline showed attached endothelial cells and minor vacuoles (arrow head in C), but also have obvious intraluminal protrusions (arrow head in C). WT lymphatic vessel displayed and intact endothelial cell layer without vacuoles and no intraluminal protrusion (D).

We observed PLN collapse shortly after CE-US evaluation of expanding PLN, which we have not previously observed in young (<5-months-old) TNF-Tg mice. To better understand this phenomenon we performed transmission electron microscopy on lymphatic vessels afferent to the PLN that received DEFINITY® and saline injections (Figure 5). The results showed endothelial cell detachment, degenerative changes to smooth muscle cells, large vacuoles and obvious intraluminal protrusions in the DEFINITY® injected tissue (Figure 5A–B). In the saline injected group, the endothelial cells displayed a normal ultrastructural morphology with minor vacuoles, and no abnormalities in the smooth muscle or connective tissue (Figure 5C) were observed compared to WT controls (Figure 5D). Thus, while CE-US may be useful for PLN phenotyping, the above findings raise concerns about potential side-effects and warrant further investigation.

**Figure 6:**
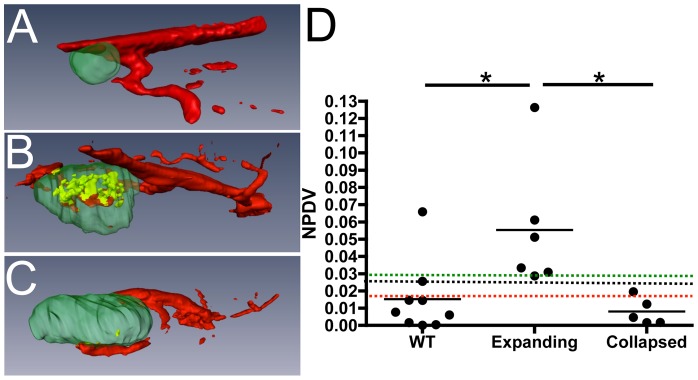
Normalized power Doppler volume is increased in expanding PLN but not in collapsed PLN. TNF-Tg and their WT littermates mice (ages 3–9 months) underwent PD-US scans and analysis as described in Figure 2. Representative 3D reconstructed images of WT (A), expanding (B), and collapsed (C) PLN (green) with their adjacent vasculature (red) are shown. The PD signal within the PLN is shown in yellow. Note the dramatic decrease in power Doppler volume (blood flow) within the collapsed PLN (B vs. C). The NPDV data are graphed to illustrate two methods that can be used to phenotype expanding vs. collapsed PLN. The first is by a strict threshold (black line, 0.025). The second is to phenotype based on percentiles; PLN falling above the 25% percentile (green line, 0.0298) are expanding, while PLN that fall below the 75% percentile are collapsed (red line, 0.01596) (n>5; *p<0.05 by Kruskal-Wallis ANVOA with Dunn's post test).

Given the aforementioned concerns about CE-US, we chose to evaluate the potential of non-invasive PD-US to phenotype the PLN as described in Figure 2, which was innocuous to the mice. The results demonstrated that NPDV was noticeably higher in TNF-Tg mice with expanding versus WT and collapsed PLN (Figure 6A–C). Moreover, we found that NPDV was significantly less in collapsed versus expanding PLN (0.008±0.003 vs. 0.553±0.007; p<0.05). This finding is consistent with the changes previously seen in LNCE [38], [39], and suggests that a decrease in blood flow in the PLN is partially responsible for PLN collapse.

**Figure 7:**
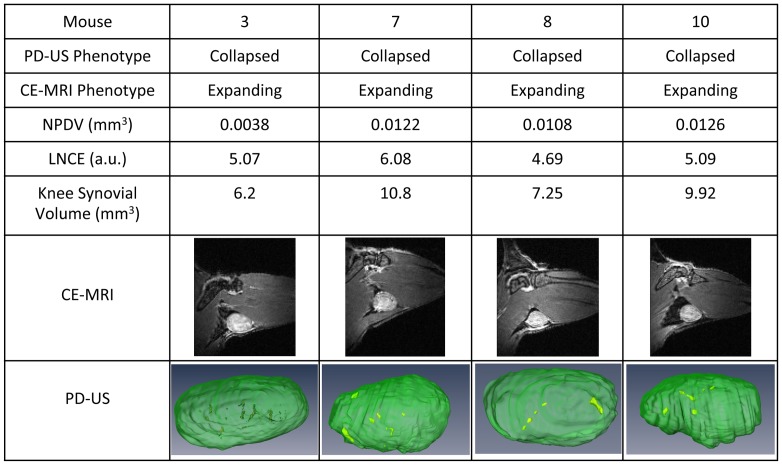
Discordant PLN phenotyping by CE-MRI and PD-US. TNF-Tg mice (ages 6–11 months) were phenotyped as collapsed by PD-US as described in Figure 6 and then underwent CE-MRI. The 4 of 12 PLN that phenotyped as collapsed via PD-US and expanding via CE-MRI are shown. Note the large synovial volumes present in the adjacent knee, a hallmark of arthritic flare and PLN collapse.

While there was general agreement between the CE-MRI and PD-US outcome measures of the 12 PLN in the study, there was disagreement over the phenotype in 4 cases (Figure 7). Of note, the LNCE of these PLN are very close to the 4.5 threshold value, suggesting that these PLN are not likely to continue expanding with increasing LNCE. Moreover, all of these PLN present with marked synovitis in the adjacent knee, a hallmark of arthritic flare and PLN collapse. Therefore, we find that PD-US correctly phenotyped all of the PLN in this cohort, and is more accurate than CE-MRI in distinguishing expanding versus collapsed PLN.

## Discussion

While arthritic flare remains an enigmatic hallmark of RA, advances in in vivo imaging have identified valuable biomarkers that may be useful towards the elucidation of arthritic flare pathophysiology and clinical management of patients. Of these biomarkers, efferent lymph nodes remain attractive but understudied due largely to the prohibitive costs associated with CE-MRI. To address this issue, here we demonstrate the feasibility of phenotyping the drainage function of murine PLN efferent to normal and arthritic joints via 3D US imaging.

In our first attempt to phenotype PLN with 3D US, we evaluated DEFINITY® as a contrast agent versus saline, and found the echogenic contrast to be critical for segmenting the lymphatic sinuses within the lymph node (Figure 1). Consistent with our hypothesis of decreased lymphatic drainage during arthritic flare [8]–[11], we found that both SVcap and PLNvol significantly increased in expanding, but not collapsed PLN following afferent footpad injection with DEFINITY® (Figure 4). Thus, CE-US is a feasible approach to phenotype murine PLN. Unfortunately, our finding that DEFINITY® administration is associated with PLN collapse and lymphatic vessel damage (Figure 5) raises concerns about the safety of this approach. Damage requires multiple DEFINITY® injections that must be subsequently imaged by US. Therefore, possible explanations for the observed lymphatic vessel damage include cavitation-induced injury. Octafluoropropane (C_3_F_8_) is imbedded in DEFINITY® lipid bubbles, which was previously reported to decrease corneal endothelial cells numbers and increase endothelial permeability [40]–[42]. In addition, performing high frequency US on C_3_F_8_ containing US contrast causes endothelial cell damage in blood vessels, including the swelling of cells, widening of the space between cells, and necrosis [43]–[45]. Based on these findings we do not support the use of CE-US for PLN phenotyping and suggest that these adverse effects need to be investigated as they relate to the clinical utilization of this test.

Our finding that PD-US is both a safe and feasible approach leads us to conclude that it is the most cost-effective means to phenotype PLN as expanding versus collapsed for prospective studies of murine inflammatory arthritis. Previously, we used strict threshold for phenotyping PLN via CE-MRI [9], [11]. A similar approach can be used for PD-US to phenotype these nodes (Figure 6D). However, due to the facts that: 1) PLN expansion is a continuum; 2) collapse is a PLN specific process that is not significantly related to PLNvol; and 3) LNCE values can occur very close to the phenotypic threshold (Figure 3C); we support a more conservative phenotyping approach that utilizes the upper and lower quartiles for each group (Figure 6D). With this approach we phenotype PLN with a NPDV greater than the 25% percentile (>0.030) as expanding PLN, whereas PLN with a NPDV less than the 75% percentile (<0.016) were considered collapsed. We also found PD-US to be more accurate for phenotyping PLN than CE-MRI in the cases of disagreement (Figure 7). Since the primary purpose of phenotyping PLN is to predict arthritic flare in the adjacent knee, and in the 4 cases of disagreement CE-MRI phenotypic failed to predict active knee synovitis, this demonstrates that PD-US is more efficient at detecting PLN collapse than CE-MRI. No formal correlation was found between NPDV and synovial volume in this cohort of mice (r^2^ = 0.1113, p>0.05). This lack of correlation could be due to a need in refinement of the method, or more likely, the fact that all of the mice that underwent both PD-US and CE-MRI were considered collapsed by PD-US and had established disease in which the synovial volume decreases due to tissue necrosis [5].

Alternative approaches to improve PLN phenotyping that were not investigated here include the use of other MRI sequences such as fat saturation [46], and diffusion tensor or T2-mapping [47], none of which have been investigated for this purpose. However, these methods still require the prohibitive cost of MRI. Therefore, by using a highly cost-effective method such as PD-US, the PLN can be more rigorously phenotyped since PLN in the middle quartiles can be subsequently imaged with longitudinal scans and studied accordingly.
